# Association between ketamine use and mortality in critically ill patients receiving mechanical ventilation: Analysis of the MIMIC-IV database

**DOI:** 10.1371/journal.pone.0320047

**Published:** 2025-03-26

**Authors:** Yuecheng Yang, Huanyu Luo, Yunkui Zhang, Zhiyong Zhao, Jun Zhang

**Affiliations:** 1 Department of Anesthesiology, Fudan University Shanghai Cancer Center, Shanghai, China,; 2 Department of Oncology, Shanghai Medical College, Fudan University, Shanghai, China,; 3 Department of Intensive Care Unit, Fudan University Shanghai Cancer Center, Shanghai, China; AOU G Martino di Messina: Azienda Ospedaliera Universitaria G Martino di Messina, Italy

## Abstract

**Objective:**

Ketamine, as a sedative, has been administered during mechanical ventilation in critically ill patients; however, its impact on survival outcomes in this patient population remains uncertain.

**Methods:**

This retrospective cohort study extracted data from the Medical Information Mart for Intensive Care (MIMIC-IV) database, version 3.0. Patients were categorized into the ketamine group and the control group based on whether ketamine was administered during mechanical ventilation. Propensity score matching was performed to adjust for demographic variables and coexisting conditions. The primary outcome was 28-day mortality. Secondary outcomes included 14-day and 90-day mortality rates, as well as hospital and ICU lengths of stay.

**Results:**

The study included a total of 8569 patients, with 330 in the ketamine group and 8239 in the control group. After propensity score matching, significant differences in mechanical ventilation duration and the proportion of patients with acute respiratory distress syndrome remained between groups. No significant differences were observed in 28-day and 90-day mortality rates between the groups. Subgroup analysis indicated that ketamine was associated with lower 14-day mortality rates among younger patients, those with acute respiratory distress syndrome, and norepinephrine users. Ketamine administration was also found to correlate with increased lengths of stay in both the hospital and ICU.

**Conclusions:**

Ketamine was more frequently selected for patients requiring prolonged mechanical ventilation. The administration of ketamine was associated with reduced 14-day but not with 28-day or 90-day mortality rates.

## Introduction

Ketamine, a non-competitive N-methyl-D-aspartate receptor antagonist, is widely used in general anesthesia due to its analgesic and sedative properties [1]. Recently, the role of ketamine in the intensive care unit (ICU) setting has garnered increasing attention [2]. Ketamine has been demonstrated to be a safe anesthetic agent for emergency intubation in the ICU [3]. Song *et al*. reported that ketamine decreased the incidence of hypotension during endoscopy [4], suggesting potential circulatory benefits during sedation. Additionally, ketamine administration may reduce opioid requirements in critically ill patients in the ICU [5].

Despite these benefits, a recent survey indicates that the majority of physicians rarely utilize ketamine for sedation or analgesia in the ICU, citing concerns about side effects [6]. A recent single-arm study identified tachycardia and sialorrhea as adverse effects associated with ketamine use in critically ill patients in the ICU [7]. Moreover, ketamine infusion has been linked to cholestatic liver injury in patients with COVID-19-associated acute respiratory distress syndrome (ARDS) [8]. However, the relationship between ketamine use and prognosis of critically ill patients in ICU has been sparsely studied. In a previous double-blind randomized controlled trial (RCT), ketamine was shown to reduce 28-day mortality in patients with mechanical ventilation (MV) (35% vs. 42%), although the difference did not reach statistical significance [9]. In another retrospective study, the use of ketamine in patients with COVID-19 was associated with improved survival rates [10]. Categorical variables necessitate a sufficiently large sample size in prospective trials. Additionally, it is well-known that extrapolating results from RCTs to clinical practice is challenging. To our knowledge, few large-scale real-world studies have been published regarding the impact of ketamine on mortality in mechanically ventilated patients in the ICU.

To address this gap, we conducted a retrospective study utilizing a public database to examine the association between ketamine administration and mortality among mechanically ventilated ICU patients. Our hypothesis was that ketamine administration would be associated with improved survival in these patients.

## Materials and methods

### Database

Data were sourced from the Medical Information Mart for Intensive Care IV (MIMIC-IV) database [11,12]. The MIMIC-IV database (Version 3.0) was released on July 23, 2024. MIMIC-IV 3.0 encompasses data from 94,458 intensive care unit admissions at the Beth Israel Deaconess Medical Center in Boston, spanning the period from 2008 to 2022. The collection of patient information and the creation of this research resource were reviewed and approved by the Institutional Review Board at Beth Israel Deaconess Medical Center, which granted a waiver of informed consent and endorsed the data sharing initiative. Therefore, the need for consent was waived in this study (https://physionet.org/content/mimiciv/3.0/). All data were anonymized and de-identified, thereby rendering ethical approval unnecessary. The authors could not identify individual participants during or after data collection, thereby ensuring patient privacy.

### Data extraction

The authors obtained data from the database on August 3, 2024. All data were extracted using Navicat software (Version 16.0). Demographic information, including age, gender, and weight, along with mechanical ventilation duration, survival status, hospital stay length, and ICU stay duration, were derived from the original dataset. Furthermore, clinical data, such as Charlson’s scores, SOFA scores, comorbidities (diabetes, myocardial infarction, congestive heart failure, dementia, and chronic pulmonary disease, which were extracted from the Charlson Comorbidity Index), vital signs (including heart rate, mean arterial pressure, SpO₂, and glucose) recorded on the first ICU day, sepsis, and norepinephrine administration were sourced from the official derived tables. The criterion for ketamine use was the administration of the drug during the period of MV. The diagnoses of acute respiratory failure (ARF), acute respiratory distress syndrome (ARDS), and severe asthma were ascertained based on the International Classification of Diseases (ICD) codes within the database, specifically: ARF (51851, 51881, J960, J9600, J9601, J9602), ARDS (J80, R0603), and severe asthma (49301, 49302, 49311, 49312, 49391, 49392, J455, J4550, J4551, J4552, J45901, J45902). Sepsis was identified in accordance with the Sepsis-3 criteria. Access to the database was granted to one author (YYC), as evidenced by certification number 53497352.

### Study design

The study was conducted as a retrospective cohort analysis. The study adhered to the STROBE guidelines. The exposure was the administration of ketamine during the MV period. Patients were categorized into the ketamine and the control groups based on exposure status. All patients receiving MV in the ICU were screened. For patients with multiple ICU admissions, only the first admission was included. The exclusion criteria were: (1) Age <  18 years; (2) MV duration <  24 hours.

To mitigate the bias from confounding factors, two propensity score-matching analyses (PSM) were performed. Model 1 adjusted for age, gender, SOFA scores, Charlson score, sepsis, ARF, ARDS, severe asthma, myocardial infarction, norepinephrine use, and congestive heart failure. Model 2 included additional adjustments for MV duration, based on Model 1.

The primary outcome was 28-day mortality. Secondary outcomes included: (1) length of ICU stay; (2) length of hospital stay; (3) in-hospital mortality; (4) 14-day mortality; (5) 90-day mortality. Additionally, subgroup analyses for mortality were performed, stratified by age, ARDS status, and vasoactive medication use.

### Statistical analysis

The Shapiro-Wilk test was employed to assess the normality of distribution. Data adhering to a normal distribution were presented as the mean ±  standard deviation. Non-normally distributed data were reported as the median (IQR). Continuous data were compared using the independent samples t-test or Mann-Whitney U test, as appropriate. Categorical data were analyzed using the Chi-square test. Outlier management was conducted using the “winsor2” package, with thresholds set at the 1st and 99th percentiles. A 1:2 PSM approach was utilized to mitigate potential confounding factors, with logistic regression employed to calculate the propensity scores. A caliper width of 0.01 was applied to ensure precise matching. Kaplan-Meier (KM) survival curves and log-rank tests were used to assess differences in survival between the two groups. The relative risk with its confidence interval (CI) was utilized to compare mortality rates across groups in subgroup analyses. Statistical significance was defined as *P* <  0.05 for all analyses. Statistical analyses were conducted using STATA, version 17.0.

## Results

**Fig 1:**
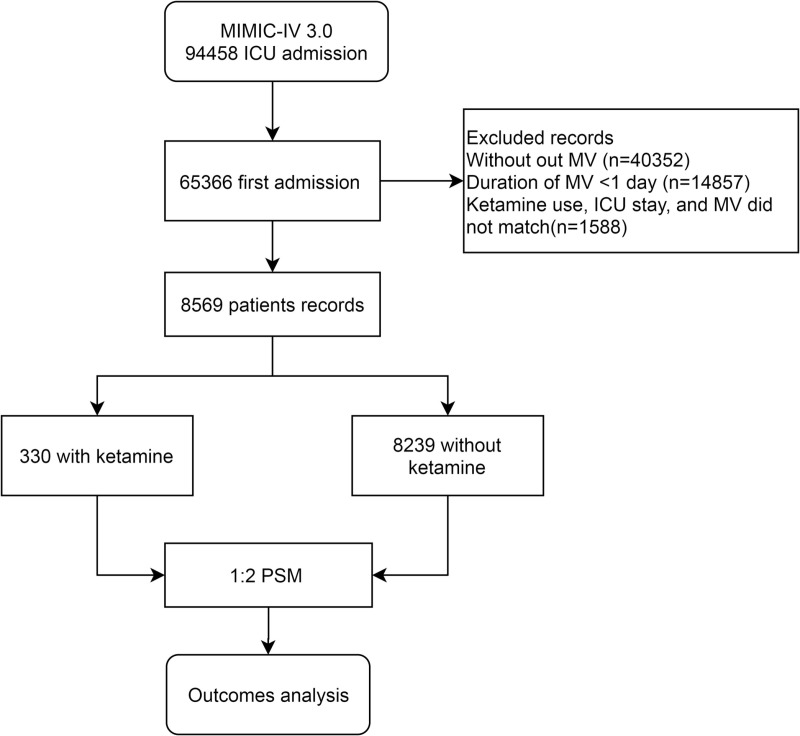
The flowchart of the study. The flowchart of the retrospective study based on MIMIC-IV database.

After applying the screening criteria, we identified 330 patients in the ketamine group and 8239 in the control group. The codes and the minimal data set are presented in the S1 File. The flowchart of this study is depicted in Fig 1. Prior to adjustment, significant differences in baseline characteristics were observed between the two groups, notably in the incidence of ARDS (40.3% vs. 3.0%, details in Table 1). Furthermore, the ketamine group exhibited a longer duration of MV. After PSM, no significant differences were observed in numerous baseline variables between the two groups, with the exception of ARDS incidence and MV duration (Table 2).

**Table 1:** Demographic data of patients with mechanical ventilation.

	Ketamine group (n = 330)	No ketamine group (n = 8239)	*P* value
Age (year)	55 (41, 62)	66 (53, 76)	<0.001
Gender (male)	222 (67.3%)	4745 (57.6%)	<0.001
Weight (Kg)	89 (77, 107)	81 (67, 98)	<0.001
SOFA scores	7 (4, 10)	7 (4, 10)	0.390
Charlson’s score	3 (1, 5)	5 (3, 7)	<0.001
Comorbidities			
Diabetes	84 (25.5%)	2436 (29.6%)	0.108
MI	28 (8.5%)	1475 (17.9%)	<0.001
CHF	55 (16.7%)	2379 (28.9%)	<0.001
CPD	85 (25.8%)	2221 (27.0%)	0.63
ARF	136 (41.2%)	4574 (55.5%)	<0.001
ARDS	133 (40.3%)	247 (3%)	<0.001
Severe Asthma	7 (2.1%)	51 (0.6%)	0.001
Dementia	3 (0.91%)	270 (3.28%)	0.016
Sepsis	303 (91.8%)	6866 (83.3%)	<0.001
Norepinephrine	199 (60.3%)	5085 (61.7%)	0.604
Duration of ventilation	5.4 (2.8, 10.5)	2.6 (1.5, 5.0)	<0.001
HR _mean_ (beat per minute)	88.5 ± 18.5	87.0 ± 17.1	0.122
MBP _mean_ (mmHg)	79.7 ± 8.8	77.8 ± 9.9	<0.001
SPO_2 mean_ (%)	96.3 (94.9, 97.8)	97.9 (96.3, 99.1)	<0.001
Glucose _mean_ (mg/dl)	138 (116, 177)	138 (117, 171)	0.79

Note: MI: myocardial infarction. CHF: congestive heart failure. CPD: chronic pulmonary disease. ARF: acute respiratory failure. ARDS: acute respiratory distress syndrome. HR: heart rate. MBP: mean blood pressure.

**Table 2:** Adjusted demographic data after propensity score matching.

	PSM Model 1			PSM Model 2		
	Ketamine group (n = 330)	Control group (n = 538)	*P value*	Ketamine group (n = 317)	Control group (n = 501)	*P value*
Age (year)	56 (42, 65)	55 (42, 66)	0.64	56 (42, 66)	56 (43, 69)	0.21
Gender (male)	222 (67.3%)	335 (62.3%)	0.13	212 (66.9%)	329 (65.7%)	0.722
Weight	89 (77, 107)	86 (71, 103)	0.06	89 (77, 106)	85 (72, 103)	0.042
SOFA score	7 (4, 10)	7 (4, 10)	0.763	7 (4, 10)	7 (4, 10)	0.771
Charlson’s score	3 (1,5)	3 (1,5)	0.2	3 (1,5)	3 (2,5)	0.053
Comorbidities						
Diabetes	84 (25.5%)	134 (24.9%)	0.86	83 (26.2%)	122 (24.4%)	0.556
MI	28 (8.5%)	56 (10.4%)	0.35	28 (8.8%)	51 (10.2%)	0.525
CHF	55 (16.7%)	110 (20.5%)	0.168	54 (17.0%)	98 (19.6%)	0.37
CPD	85 (25.8%)	118 (21.9%)	0.196	80 (25.2%)	122 (24.4%)	0.775
ARF	136 (41.2%)	256 (47.6%)	0.067	136 (42.9%)	249 (49.7%)	0.058
ARDS	133 (40.3%)	162 (30.1%)	0.002	121 (38.2%)	140 (27.9%)	0.002
Severe Asthma	7 (2.1%)	10 (1.9%)	0.786	7 (2.2%)	10 (2%)	0.836
Dementia	3 (0.91%)	12 (2.23%)	0.147	3 (0.95%)	11 (2.2%)	0.18
Sepsis	303 (91.8%)	484 (89.6%)	0.28	290 (91.5%)	439 (87.6%)	0.08
Norepinephrine	199 (60.3%)	327 (60.8%)	0.89	193 (60.9%)	309 (61.7%)	0.82
Duration of ventilation	5.4 (2.8, 10.5)	3.0 (1.8, 5.4)	<0.001	5.2 (2.7, 9.5)	3.5 (1.9, 7.3)	<0.001
HR _mean_ (beat per minute)	88.5 ± 18.5	89.9 ± 17.4	0.258	88.3 ± 18.6	89.9 ± 17.5	0.229
MBP _mean_ (mmHg)	79.7 ± 8.8	78.6 ± 9.8	0.125	79.6 ± 8.7	78.9 ± 9.9	0.3
SPo2 _mean_ (%)	96.3 (94.9, 97.8)	97.2 (95.5, 98.9)	<0.001	96.4 (95, 97.8)	97.0 (95.2, 98.7)	<0.001
Glucose _mean_ (mg/dl)	138 (116, 177)	137 (116, 173)	0.625	139 (116, 177)	140 (117, 175)	0.9

Note: MI: myocardial infarction. CHF: congestive heart failure. ARF: acute respiratory failure. ARDS: acute respiratory distress syndrome. HR: heart rate. MBP: mean blood pressure. CPD: chronic pulmonary disease.

In Model 1, the matching variables included age, gender, MI, CHF, ARF, ARDS, Charlson score, SOFA, and Sepsis. In Model 2, MV duration was added to the matching variables.

**Fig 2:**
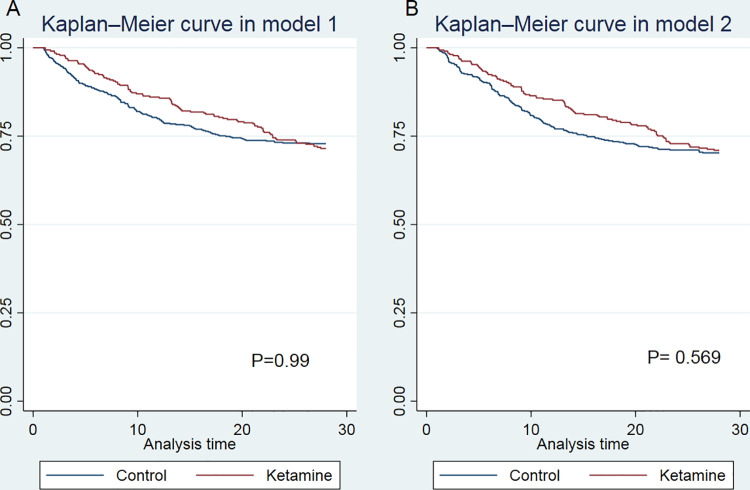
Kaplan–Meier survival curves depicting 28-day mortality following propensity score matching. (A) Survival curves of patients following adjustments for demographic data and comorbid conditions. Model 1: Adjusted variables comprised age, sex, ARF, ARDS, severe asthma, myocardial infarction, congestive heart failure, Charlson Comorbidity Index, SOFA scores, and sepsis. (B) Survival curves of patients following adjustments for demographic data, comorbid conditions, and mechanical ventilation duration. Model 2: Adjusted variables included mechanical ventilation duration in addition to those in Model 1.

The outcomes are summarized in Table 3. No significant differences were noted in 28-day, in-hospital, and 90-day mortalities between the groups. Nonetheless, the use of ketamine was associated with a reduction in 14-day mortality in PSM Model 2 (17.7% vs. 24.2%, *P* = 0.028). The 28-day mortality survival outcomes are illustrated in Fig 2. Additionally, in both models, the ketamine group demonstrated prolonged lengths of stay in both the hospital and ICU compared with the control group (*P* < 0.001).

**Table 3:** The outcomes data in the adjusted cohort.

	PSM model 1			PSM model 2		
	Ketamine (n = 330)	No ketamine (n = 538)	P value	Ketamine (n = 317)	No ketamine (n = 501)	P value
Hospital stay (day)	21.8 (13.2, 35.6)	14.4 (8.5, 23.9)	<0.001	20.9 (12.5, 33.8)	13.8 (8.2, 23.9)	<0.001
ICU stay (day)	14.0 (7.1, 23.2)	7.9 (4.4, 13.9)	<0.001	13.6 (6.9, 22.0)	8.7 (4.5, 14.5)	<0.001
Post-ventilation ICU stay	3.7 (1.2, 10.0)	2.3 (1.0, 5.2)	<0.001	3.5 (1.2, 9.9)	1.9 (0.7, 4.6)	<0.001
In-hospital mortality	95 (28.8%)	138 (25.7%)	0.311	90 (28.4%)	149 (29.7%)	0.679
14-day mortality	56 (17%)	117 (21.8%)	0.087	56 (17.7%)	121 (24.2%)	0.028
28-day mortality	94 (28.5%)	146 (27.1%)	0.667	92 (29.0%)	149 (29.7%)	0.826
90-day mortality	110 (33.3%)	172 (32.0%)	0.677	105 (33.1%)	183 (36.5%)	0.321

**Fig 3:**
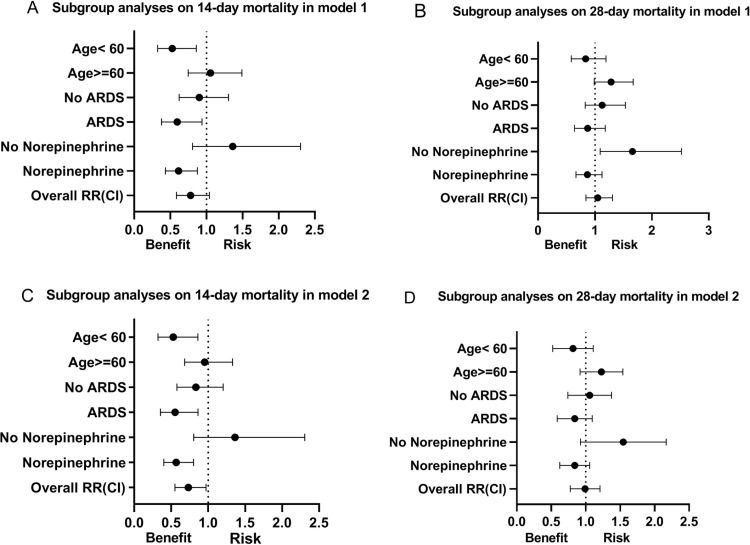
Forest plot displaying the subgroup analyses of mortality risk. Figure legend: The overall and subgroup analyses of mortality are presented as risk ratios with their 95% confidence intervals. A risk ratio less than 1 indicates that ketamine is associated with reduced mortality compared with the control group. (A) Subgroup analysis of 14-day mortality in PSM Model 1. (B) Subgroup analysis of 28-day mortality in PSM Model 1. (C) Subgroup analysis of 14-day mortality in PSM Model 2. (D) Subgroup analysis of 28-day mortality in PSM Model 2.

The relative risk of mortality between the groups is presented in Fig 3. Ketamine was associated with improved 14-day mortality in Model 2 [RR: 0.73 (95% CI, 0.55 to 0.97), *P* = 0.028]. Subgroup analyses revealed that younger age, ARDS, and norepinephrine use were associated with a decreased risk of 14-day mortality across both models. Nonetheless, for 28-day mortality, these subgroups did not exhibit survival benefits in either model.

## Discussion

In this study, we found that ketamine was not associated with a reduction in in-hospital mortality among critically ill patients requiring MV. Conversely, the study revealed that ketamine was associated with prolonged in-hospital and ICU stays.

The impact of ketamine on the duration of MV in the ICU remains controversial. Previous RCTs indicated that ketamine infusion did not extend the duration of MV [9,13]. A retrospective analysis suggested that ketamine was associated with an extended length of hospital stay for COVID-19 patients with ARDS (by 7 days) [10]. However, in another study targeting COVID-19 patients, the use of ketamine during MV was associated with a reduced length of hospital stay [14]. In our study, the duration of MV was significantly longer in patients receiving ketamine, indicating that physicians preferred to use ketamine in patients who were expected to require relatively long-term MV. Furthermore, the length of post-MV ICU stay was also significantly longer in the ketamine group. The findings from RCTs may not be readily generalizable to clinical practice, given the complex and critical disease progression observed in ICU patients. Therefore, despite confounding bias, ketamine was associated with longer hospital and ICU stays. The prolonged hospital and ICU stays may be partially attributable to the neurocognitive effects of ketamine [10]. Blecha *et al.* also found that ketamine was associated with an increased risk of long-term psychiatric symptoms after hospital discharge [15]. Regrettably, the MIMIC-IV database did not include data on long-term psychiatric symptoms.

Ketamine did not cause significant alterations in blood pressure, heart rate, or vascular resistance when compared with other sedative agents [16,17]. In prior prospective studies, ketamine did not demonstrate survival benefits for patients undergoing MV [9,13]. Alwakeel *et al*. proposed that in COVID-19 patients requiring MV support, the impact of sedative agents on patient mortality was almost negligible [18]. However, in a retrospective study, ketamine was associated with decreased mortality in patients with MV [10]. Additionally, a recent study demonstrated that ketamine, compared with etomidate, could reduce the in-hospital mortality rate of MV patients [19]. This also reflects the potential benefit of ketamine in reducing mortality. An animal study showed that ketamine can attenuate systemic inflammation and multi-organ injury in mice [20]. In the present study, we only found survival benefits in 14-day mortality. However, the survival benefits in in-hospital mortality and 90-day mortality were limited. Our study suggests that the use of ketamine in MV patients does not provide a significant benefit in terms of overall survival.

Subgroup analyses revealed that patients with ARDS in the ketamine group exhibited a relatively higher survival rate at 14 days in the ICU. A plausible explanation is that ketamine may reduce airway resistance and the work of breathing [21]. An animal model study demonstrated that ketamine mitigated lung injury induced by MV through modulation of inflammatory factor expression [22]. Another basic research study also found that esketamine can alleviate ferroptosis-mediated acute lung injury [23]. Xu *et al*. reported that ketamine might attenuate high mobility group box protein 1-induced acute lung injury by regulating the Toll-like receptor 4 signaling pathway [24]. Clinically, a previous meta-analysis showed that perioperative ketamine inhibited the IL-6 inflammatory response [25]. It is well-established that inflammatory factors, including IL-6, are intimately linked to the pathogenesis and prognosis of ARDS [26]. This finding also elucidates why the prevalence of ARDS was notably higher in the ketamine group prior to PSM. The subgroup analyses further revealed that patients requiring norepinephrine exhibited enhanced survival outcomes at 14-day mortality. A likely explanation is that ketamine did not induce hemodynamic fluctuations [17]. Nonetheless, these survival improvements were not sustained at 28-day.

A strength of this study is its relatively larger sample size compared with prior studies [9,13,27]. The sample size in a past single-arm study is relatively large [5]; however, the survival benefit of ketamine cannot be conclusively determined from that study. Survival results derived from cohort studies with large sample sizes are more robust. Our study contributes clinical evidence from real-world data regarding the impact of ketamine on patients with MV. Furthermore, additional subgroup analyses indicated a potential benefit of ketamine for patients diagnosed with ARDS. To minimize the impact of confounding factors on survival data, we employed two PSM models to reduce bias in comorbidity and duration of MV between the groups. Both PSM models suggest that ketamine is potentially associated with improved 14-day survival rates in patients with ARDS.

This study had several limitations. Firstly, the retrospective nature of the study may have introduced unmeasured biases. Secondly, despite PSM, significant differences in the proportions of patients with a diagnosis of ARDS and the duration of MV remained between the groups. Thirdly, the sample size of the ketamine group was relatively small, given the large size of the MIMIC-IV database. Consequently, a 1:2 matching ratio was utilized to ensure adequate comparability.

## Conclusion

In this retrospective study, we observed that younger patients with ARDS may benefit from ketamine use in terms of 14-day mortality. However, these benefits were not observed in longer-term survival outcomes. Overall, the effect of ketamine on mortality appears to be limited. In clinical practice, the preferential use of ketamine for patients with extended duration of MV may introduce bias into the analysis of survival outcomes. This study contributes real-world evidence to the understanding of ketamine’s impact on mortality in the ICU. Further prospective studies are warranted to elucidate the impact of ketamine use on mortality in critically ill patients with ARDS.

## Supporting information

S1 file The codes and minimal data set of the study. (ZIP)
