# Supplementary material for: Association between ketamine use and mortality in critically ill patients receiving mechanical ventilation: Analysis of the MIMIC-IV database
Source: PLoS One. 2025 Mar 26;20(3):e0320047. doi: 10.1371/journal.pone.0320047 (PMC11940653; doi:10.1371/journal.pone.0320047)
Supplement: S1 file — (ZIP) [file pone.0320047.s001.zip › S1 file/raw data/raw data code for NAVICAT.docx]

**Code for Navicat**

SELECT * FROM mimiciv_derived.ventilation where lower(ventilation_status)='invasivevent'

with ket as

(select subject_id, hadm_id, starttime, stoptime, drug, case when lower(drug)='ketamine' then 1 else 0 end as ket_use from mimiciv_hosp.prescriptions group by subject_id, hadm_id, starttime, stoptime, drug order by prescriptions. subject_id)

select * from ket where ket_use=1

select * from mimiciv_derived.icustay_detail where icustay_seq=1

select * from mimiciv_derived.first_day_sofa

select * from mimiciv_derived.first_day_weight

select * from mimiciv_derived.charlson

select * from mimiciv_derived.first_day_vitalsign

select * from mimiciv_derived.sepsis3

select * from mimiciv_derived.norepinephrine_equivalent_dose

select * from mimiciv_derived.first_day_gcs

code for ARF: 51851 51881 J960 J9600 J9601 J9602

with arff as

(select subject_id, hadm_id, seq_num, icd_code, icd_version, case when icd_code='51851' then 1

when icd_code= '51881' then 1

when icd_code= 'J960' then 1

when icd_code= 'J9600' then 1

when icd_code= 'J9601' then 1

when icd_code= 'J9602' then 1 else 0 end as arf from mimiciv_hosp.diagnoses_icd group by subject_id, hadm_id, seq_num, icd_code, icd_version order by subject_id)

select * from arff where arf=1

Code for ARDS: J80 R0603

with ardss as

(select subject_id, hadm_id, seq_num, icd_code, icd_version, case when icd_code='J80' then 1

when icd_code= 'R0603' then 1 else 0 end as ards from mimiciv_hosp.diagnoses_icd group by subject_id, hadm_id, seq_num, icd_code, icd_version order by subject_id)

select * from ardss where ards=1

Code for asthma: 49301  49302 49311 49312 49391 49392 J455 J4550 J4551 J4552 J45901 J45902
