# Supplementary material for: Association between ketamine use and mortality in critically ill patients receiving mechanical ventilation: Analysis of the MIMIC-IV database
Source: PLoS One. 2025 Mar 26;20(3):e0320047. doi: 10.1371/journal.pone.0320047 (PMC11940653; doi:10.1371/journal.pone.0320047)
Supplement: S1 file — (ZIP) [file pone.0320047.s001.zip › S1 file/raw data/raw data code for STATA.docx]

duplicates report stay_id

Duplicates in terms of stay_id

duplicates report subject_id

Duplicates in terms of subject_id

. sort subject_id

. keep if hospstay_seq==1

(19,876 observations deleted)

. count

65,366

. save "C:\Users\hasee\Desktop\mimic\icu_stay.dta"

file C:\Users\hasee\Desktop\mimic\icu_stay.dta saved

. use "C:\Users\hasee\Desktop\mimic\icu_stay.dta"

. log on

(log already on)

. replace gender="0" if gender=="F"

(28,646 real changes made)

. replace gender="1" if gender=="M"

(36,720 real changes made)

. destring gender

must specify either generate or replace option

r(198);

. destring gender, replace

gender: all characters numeric; replaced as byte

. gen admittime_1=clock( admittime,"DMYhms")/3600/1000

. gen dischtime_1=clock( dischtime,"DMYhms")/3600/1000

. rename admission_age age

. gen icu_intime_1=clock( icu_intime,"DMYhms")/3600/1000

. gen icu_outtime_1=clock( icu_outtime,"DMYhms")/3600/1000

(12 missing values generated)

. gen dod_1=clock(dod,"DMY")/3600/1000

(43,424 missing values generated)

. drop icustay_seq

. tab first_icu_stay

. drop first_icu_stay

. su age,d

. count if age<18

0

. tab hospstay_seq

. drop hospstay_seq first_hosp_stay

. drop dod admittime dischtime icu_intime icu_outtime

. save "C:\Users\hasee\Desktop\mimic\icu_stay.dta", replace

file C:\Users\hasee\Desktop\mimic\icu_stay.dta saved

. gen death_in_icu=1 if icu_outtime_1> dod_1

(60,320 missing values generated)

. replace death_in_icu=0 if death_in_icu==.

(60,320 real changes made)

. gen death_time= dod_1- icu_intime_1

(43,424 missing values generated)

. replace death_time= death_time/24

(21,928 real changes made)

. save "C:\Users\hasee\Desktop\mimic\icu_stay.dta", replace

file C:\Users\hasee\Desktop\mimic\icu_stay.dta saved

. drop if death_time<1

(1,828 observations deleted)

. drop if los_icu<1

(12,127 observations deleted)

. count

51,411

. save "C:\Users\hasee\Desktop\mimic\icu_stay.dta", replace

file C:\Users\hasee\Desktop\mimic\icu_stay.dta saved

. drop race

. save "C:\Users\hasee\Desktop\mimic\icu_stay.dta", replace

file C:\Users\hasee\Desktop\mimic\icu_stay.dta saved

. duplicates report subject_id

. save "C:\Users\hasee\Desktop\mimic\icu_stay.dta", replace

file C:\Users\hasee\Desktop\mimic\icu_stay.dta saved

. exit, clear

---------------------------------------------------------------------------------------------------------------------------

name: <unnamed>

log: C:\Users\hasee\Desktop\mimic\氯胺酮.smcl

log type: smcl

opened on: 30 Jul 2024, 13:47:05

. duplicates report stay_id

. duplicates report subject_id

. sort subject_id

. drop respiration coagulation liver cardiovascular cns renal

. save "C:\Users\hasee\Desktop\mimic\firstday_sofa.dta"

file C:\Users\hasee\Desktop\mimic\firstday_sofa.dta saved

. clear

. import delimited "C:\Users\hasee\Desktop\mimic\csv原始文件\所有氯胺酮.csv"

(encoding automatically selected: ISO-8859-9)

(6 vars, 4,264 obs)

. drop drug

. gen ket_starttime_1=clock( starttime,"DMYhms")/3600/1000

. gen ket_stoptime_1=clock( stoptime,"DMYhms")/3600/1000

(3 missing values generated)

. rename ket_starttime_1 ket_starttime

. rename ket_stoptime_1 ket_stoptime

. drop starttime stoptime

. save "C:\Users\hasee\Desktop\mimic\氯胺酮.dta"

file C:\Users\hasee\Desktop\mimic\氯胺酮.dta saved

. save "C:\Users\hasee\Desktop\mimic\氯胺酮.dta", replace

file C:\Users\hasee\Desktop\mimic\氯胺酮.dta saved

. use "C:\Users\hasee\Desktop\mimic\icu_stay.dta"

. duplicates report hadm_id

. merge 1:m hadm_id using C:\Users\hasee\Desktop\mimic\氯胺酮.dta

. drop if _merge==2

(2,179 observations deleted)

. gen ket_icu=1 if ket_starttime > icu_intime_1

(91 missing values generated)

. drop ket_icu

. gen ket-icu= ket_starttime- icu_intime_1

invalid syntax

r(198);

. gen ket_icu_time= ket_starttime- icu_intime_1

(50,593 missing values generated)

. gen ket_icuout_time= icu_outtime_1- ket_stoptime

(50,608 missing values generated)

. drop ket_icuout_time

. gen ket_icutime_1= icu_outtime_1- ket_starttime

(50,607 missing values generated)

. drop _merge

. drop ket_icu_time ket_icutime_1

. save "C:\Users\hasee\Desktop\mimic\icu_stay.dta", replace

file C:\Users\hasee\Desktop\mimic\icu_stay.dta saved

. exit, clear

---------------------------------------------------------------------------------------------------------------------------

name: <unnamed>

log: C:\Users\hasee\Desktop\mimic\氯胺酮.smcl

log type: smcl

opened on: 30 Jul 2024, 14:43:58

. count if ven_start< icu_intime_1

2,094

. count if ven_start> icu_outtime_1

9

. count

30,152

. drop if ven_start< icu_intime_1

(2,094 observations deleted)

. drop if ven_start> icu_outtime_1

(9 observations deleted)

. count

28,049

. gen ven_duration= ven_end- ven_start

. replace ven_duration= ven_duration/24

(28,044 real changes made)

. save "C:\Users\hasee\Desktop\mimic\merge_呼吸机_icustay.dta", replace

file C:\Users\hasee\Desktop\mimic\merge_呼吸机_icustay.dta saved

. duplicates report stay_id

. sort stay_id ven_start

. save "C:\Users\hasee\Desktop\mimic\merge_呼吸机_icustay.dta", replace

file C:\Users\hasee\Desktop\mimic\merge_呼吸机_icustay.dta saved

. duplicates report hadm_id

. merge m:m hadm_id using C:\Users\hasee\Desktop\mimic\氯胺酮.dta

. drop if _merge==2

(2,725 observations deleted)

. save "C:\Users\hasee\Desktop\mimic\merge_icu呼吸机氯胺酮.dta"

file C:\Users\hasee\Desktop\mimic\merge_icu呼吸机氯胺酮.dta saved

. drop _merge

. count if ket_use==1

2,011

. count if ket_starttime< icu_intime_1

39

. count if ket_starttime> ven_end

27,234

. count if ket_starttime< ven_start

749

. gen ket_icu_starttime= ket_starttime- ven_start

(26,667 missing values generated)

. sort ket_icu_starttime

. replace ket_icu_starttime= ket_icu_starttime/24

(1,991 real changes made)

. drop if ket_icu_starttime<0

(749 observations deleted)

. drop ket_icu_starttime

. gen ket_icu_ven= ven_end- ket_starttime

(26,667 missing values generated)

. sort ket_icu_ven

. gen ket_icu_ven= ket_icu_ven/24

variable ket_icu_ven already defined

r(110);

. replace ket_icu_ven= ket_icu_ven/24

(1,254 real changes made)

. drop if ket_icu_ven<0

(567 observations deleted)

. count if ket_use==1

695

. drop ket_icu_ven

. save "C:\Users\hasee\Desktop\mimic\merge_icu呼吸机氯胺酮.dta", replace

file C:\Users\hasee\Desktop\mimic\merge_icu呼吸机氯胺酮.dta saved

. sort stay_id ket_use

. sort stay_id ket_starttime

. duplicates drop stay_id,force

Duplicates in terms of stay_id

(6,435 observations deleted)

. count if ket_use==1

395

. duplicates report stay_id

. save "C:\Users\hasee\Desktop\mimic\merge_icu呼吸机氯胺酮.dta", replace

file C:\Users\hasee\Desktop\mimic\merge_icu呼吸机氯胺酮.dta saved

. duplicates report subject_id

. merge 1:1 hadm_id using C:\Users\hasee\Desktop\mimic\察尔森评分.dta

. keep if _merge==3

(525,101 observations deleted)

. save "C:\Users\hasee\Desktop\mimic\ICU呼吸机氯胺酮开始合并症.dta"

file C:\Users\hasee\Desktop\mimic\ICU呼吸机氯胺酮开始合并症.dta saved

. drop _merge

. duplicates report stay_id

. merge 1:1 stay_id using C:\Users\hasee\Desktop\mimic\firstday_sofa.dta

. keep if _merge==3

(73,531 observations deleted)

. drop _merge

. save "C:\Users\hasee\Desktop\mimic\ICU呼吸机氯胺酮开始合并症.dta", replace

file C:\Users\hasee\Desktop\mimic\ICU呼吸机氯胺酮开始合并症.dta saved

. merge 1:1 stay_id using C:\Users\hasee\Desktop\mimic\第一天生命体征.dta

. keep if _merge==3

(73,531 observations deleted)

. drop _merge

. save "C:\Users\hasee\Desktop\mimic\ICU呼吸机氯胺酮开始合并症.dta", replace

file C:\Users\hasee\Desktop\mimic\ICU呼吸机氯胺酮开始合并症.dta saved

. merge 1:1 stay_id using "C:\Users\hasee\Desktop\mimic\sepsis.dta"

. drop if _merge==2

(27,087 observations deleted)

. replace sepsis3=0 if sepsis3==.

(6,719 real changes made)

. save "C:\Users\hasee\Desktop\mimic\ICU呼吸机氯胺酮开始合并症.dta", replace

file C:\Users\hasee\Desktop\mimic\ICU呼吸机氯胺酮开始合并症.dta saved

. drop _merge

. save "C:\Users\hasee\Desktop\mimic\ICU呼吸机氯胺酮开始合并症.dta", replace

file C:\Users\hasee\Desktop\mimic\ICU呼吸机氯胺酮开始合并症.dta saved

. gen death_hosp dischtime_1 =1 if dod_1< dischtime_1

variable dischtime_1 already defined

r(110);

. gen death_hosp=1 if dod_1 < dischtime_1

(17,964 missing values generated)

. replace death_hosp=0 if death_hosp==.

(17,964 real changes made)

. save "C:\Users\hasee\Desktop\mimic\ICU呼吸机氯胺酮开始合并症.dta", replace

file C:\Users\hasee\Desktop\mimic\ICU呼吸机氯胺酮开始合并症.dta saved

. gen death_28=1 if death_time <=28

(17,494 missing values generated)

. replace death_28=0 if death_28==.

(17,494 real changes made)

. gen death_90=1 if death_time <=90

(16,582 missing values generated)

. replace death_90=0 if death_90==.

(16,582 real changes made)

. save "C:\Users\hasee\Desktop\mimic\ICU呼吸机氯胺酮开始合并症.dta", replace

file C:\Users\hasee\Desktop\mimic\ICU呼吸机氯胺酮开始合并症.dta saved

. stset death_time, failure(death_90)

. sts graph, by(ket_use) tmax(90)

Failure _d: death_90

Analysis time _t: death_time

. replace ket_use=0 if ket_use==.

(20,532 real changes made)

. save "C:\Users\hasee\Desktop\mimic\ICU呼吸机氯胺酮开始合并症.dta", replace

file C:\Users\hasee\Desktop\mimic\ICU呼吸机氯胺酮开始合并症.dta saved

. tab ket_use

. save "C:\Users\hasee\Desktop\mimic\ICU呼吸机氯胺酮开始合并症.dta", replace

file C:\Users\hasee\Desktop\mimic\ICU呼吸机氯胺酮开始合并症.dta saved

. psmatch2 ket_use gender age sepsis3 charlson_comorbidity_index sofa, outcome( death_90 ) logit noreplacement n(1) caliper

> (0.02)

. save "C:\Users\hasee\Desktop\mimic\psm 后试运行.dta"

file C:\Users\hasee\Desktop\mimic\psm 后试运行.dta saved

. keep if _weight==1

(20,137 observations deleted)

. tab ket_use

. sts graph, by(ket_use) tmax(90)

Failure _d: death_90

Analysis time _t: death_time

. sts test ket_use

Failure _d: death_90

Analysis time _t: death_time

Equality of survivor functions

Log-rank test

. save "C:\Users\hasee\Desktop\mimic\psm 后试运行.dta", replace

file C:\Users\hasee\Desktop\mimic\psm 后试运行.dta saved

. sts graph, by(ket_use) tmax(90)

Failure _d: death_90

Analysis time _t: death_time

. tab ket_use death_hosp, row chi2

. tab ket_use death_28, row chi2

. tab ket_use death_90, row chi2

. rename ket_use group

. tab group sepsis3, chi2

. ttest charlson_comorbidity_index, by(group) unequal

. tab group myocardial_infarct, row chi2

. save "C:\Users\hasee\Desktop\mimic\psm 后试运行.dta", replace

file C:\Users\hasee\Desktop\mimic\psm 后试运行.dta saved

. use "C:\Users\hasee\Desktop\mimic\ICU呼吸机氯胺酮开始合并症.dta", clear

. rename ket_use group

. tab group

. sk test age

. sktest age

. histogram age

(bin=43, start=18.02441, width=1.9043951)

. tabstat age, by(group) stat(P25, P50, P75)

. ranksum age, by(group)

. tab group gender, row chi2

. ranksum sofa, by(group)

. tabstat sofa charlson_comorbidity_index, by(group)

. tabstat sofa charlson_comorbidity_index, by(group) stat(P25, P50, P75)

. ranksum charlson_comorbidity_index, by(group)

. gen diabetes= diabetes_without_cc+ diabetes_with_cc

. replace diabetes=1 if diabetes!=0

(697 real changes made)

. tab group diabetes, row chi2

. tab group myocardial_infarct, row chi2

. tab group congestive_heart_failure, row chi2

. tab group dementia, row chi2

. tab group sepsis3, row chi2

. histogram heart_rate_mean mbp_mean temperature_mean glucose_mean

too many variables specified

r(103);

. histogram heart_rate_mean

(bin=43, start=41, width=2.8239203)

. ttest heart_rate_mean, by(group)

Pr(T < t) = 0.0000 Pr(|T| > |t|) = 0.0000 Pr(T > t) = 1.0000

. histogram mbp_mean,by(group)

. ttest mbp_mean,by(group)

. histogram spo2_mean, by(group)

. tabstat spo2_mean,by(group) stat(P25, P50, P75)

. ranksum spo2_mean, by(group)

. histogram glucose_mean, by(group)

. tabstat glucose_mean, by(group) stat(P25, P50, P75)

Summary for variables: glucose_mean

Group variable: group

group | p25 p50 p75

---------+------------------------------

0 | 118.0556 131.6667 153.6429

1 | 115.25 135.8333 170.4167

---------+------------------------------

Total | 118 131.7222 154

----------------------------------------

. ranksum glucose_mean, by(group)

. save "C:\Users\hasee\Desktop\mimic\ICU呼吸机氯胺酮开始合并症.dta", replace

file C:\Users\hasee\Desktop\mimic\ICU呼吸机氯胺酮开始合并症.dta saved

. merge 1:1 stay_id using "C:\Users\hasee\Desktop\mimic\firstday_weight.dta"

. keep if _merge==3

(73,531 observations deleted)

. drop _merge

. save "C:\Users\hasee\Desktop\mimic\ICU呼吸机氯胺酮开始合并症.dta", replace

file C:\Users\hasee\Desktop\mimic\ICU呼吸机氯胺酮开始合并症.dta saved

. histogram weight, by(group)

. ttest weight, by(group) unequal

. scatter weight

too few variables specified

r(102);

. su weight, d

. twoway scatter weight age

. ssc install winsor2, replace

checking winsor2 consistency and verifying not already installed...

all files already exist and are up to date.

. winsor2 weight, cut(1,99)

. ttest weight, by(group) unequal

. ttest weight_w , by(group) unequal

. sort weight

. drop weight

. rename weight_w weight

. save "C:\Users\hasee\Desktop\mimic\ICU呼吸机氯胺酮开始合并症.dta", replace

file C:\Users\hasee\Desktop\mimic\ICU呼吸机氯胺酮开始合并症.dta saved

. count if weight==.

60

. su weight

. replace weight=85 if weight==.

(60 real changes made)

. save "C:\Users\hasee\Desktop\mimic\ICU呼吸机氯胺酮开始合并症.dta", replace

file C:\Users\hasee\Desktop\mimic\ICU呼吸机氯胺酮开始合并症.dta saved

. ttest weight, by(group) unequal

.

.

. save "C:\Users\hasee\Desktop\mimic\ICU呼吸机氯胺酮开始合并症.dta", replace

file C:\Users\hasee\Desktop\mimic\ICU呼吸机氯胺酮开始合并症.dta saved

. merge 1:m stay_id using "C:\Users\hasee\Desktop\mimic\去甲肾数据.dta"

. save "C:\Users\hasee\Desktop\mimic\完整数据+去甲.dta"

file C:\Users\hasee\Desktop\mimic\完整数据+去甲.dta saved

. sort norepinephrine_equivalent_dose

. gen neropine_use=0 if norepinephrine_equivalent_dose==.

(783,613 missing values generated)

. replace neropine_use=1 if neropine_use==.

(783,613 real changes made)

. drop _merge

. gen ne_vent_start= ne_starttime- icu_intime_1

(389,026 missing values generated)

. drop if icu_intime_1==.

(380,347 observations deleted)

. count

411,945

. sort ne_vent_start

. replace ne_vent_start= ne_vent_start/24

(402,921 real changes made)

. replace neropine_use=0 if ne_vent_start<0

(294 real changes made)

. drop ne_vent_start

. gen ne_vent_end= icu_outtime_1- ne_starttime

(9,393 missing values generated)

. sort ne_vent_end

. replace neropine_use=0 if ne_vent_end<0

(11 real changes made)

. bys stay_id: egen ne_max=max(neropine_use)

. gsort -stay_id - ne_max

. duplicates drop stay_id, force

Duplicates in terms of stay_id

(391,018 observations deleted)

. tab group

. save "C:\Users\hasee\Desktop\mimic\完整数据+去甲.dta", replace

file C:\Users\hasee\Desktop\mimic\完整数据+去甲.dta saved

. replace neropine_use= ne_max

(5 real changes made)

. drop ne_max

. save "C:\Users\hasee\Desktop\mimic\完整数据+去甲.dta", replace

file C:\Users\hasee\Desktop\mimic\完整数据+去甲.dta saved

. tab group neropine_use, row chi2
